# Pediatric Bullous Impetigo: A Case Report and Literature Review

**DOI:** 10.7759/cureus.99351

**Published:** 2025-12-16

**Authors:** Noor Altaho, Reem AlQusaimi

**Affiliations:** 1 Pediatrics, Al-Amiri Hospital, Kuwait City, KWT; 2 Medicine and Surgery, Kuwait Institute for Medical Specialization, Kuwait City, KWT

**Keywords:** bullous impetigo, impetigo, skin infection, staphylococcus aureus, superficial blistering disorder

## Abstract

Impetigo is a common superficial bacterial skin infection characterized by vesiculobullous or crusted lesions. Bullous impetigo presents with flaccid bullae that rupture easily, leaving superficial erosions and thin crusting. Unlike staphylococcal scalded skin syndrome, the blistering in bullous impetigo remains localized because the exfoliative toxins act within the skin rather than disseminating systemically.

We report the case of a previously healthy two-year-old girl who presented with gradually progressive pruritic erosions and honey-colored crusts localized to the right lower limb. Two siblings had similar but milder perioral lesions, which were briefly assessed clinically and considered consistent with early, limited impetigo, suggesting a possible small household cluster. Clinical evaluation supported a diagnosis of bullous impetigo. She received intravenous cefazolin in addition to topical fusidic acid and emollients, leading to steady improvement in pain, pruritus, and lesion appearance.

This case underscores the importance of early recognition of bullous impetigo and highlights practical considerations in its diagnosis and treatment, particularly in pediatric patients. A clear clinical approach can help avoid misdiagnosis, reduce unnecessary investigations, and guide appropriate antimicrobial therapy.

## Introduction

Impetigo is a highly contagious superficial bacterial infection of the epidermis and represents one of the most common childhood skin diseases worldwide. The global point prevalence is estimated at more than 140 million cases, with the highest incidence occurring in children aged two to five years. Impetigo typically thrives in warm, humid environments and is associated with risk factors such as poor hygiene, overcrowding, low socioeconomic status, and coexisting skin conditions such as scabies [1].

Two classic clinical forms are recognized: non-bullous and bullous impetigo. Non-bullous impetigo accounts for approximately 70% of cases and is caused by *Staphylococcus aureus*, *Streptococcus pyogenes*, or both. Lesions often arise at sites of minor trauma and evolve from small vesicles to characteristic honey-colored crusted erosions. Bullous impetigo, by contrast, is exclusively due to exfoliative toxin-producing *S. aureus*, which induces intraepidermal cleavage and the formation of flaccid bullae. These bullae rupture easily, leaving shallow erosions bordered by a collarette of scale [2].

Although usually mild and self-limiting, impetigo warrants prompt treatment to reduce transmission and prevent complications. Here, we report a case of bullous impetigo in a two-year-old girl, followed by a brief review of diagnostic and therapeutic considerations in pediatric impetigo.

## Case presentation

**Figure 1:**
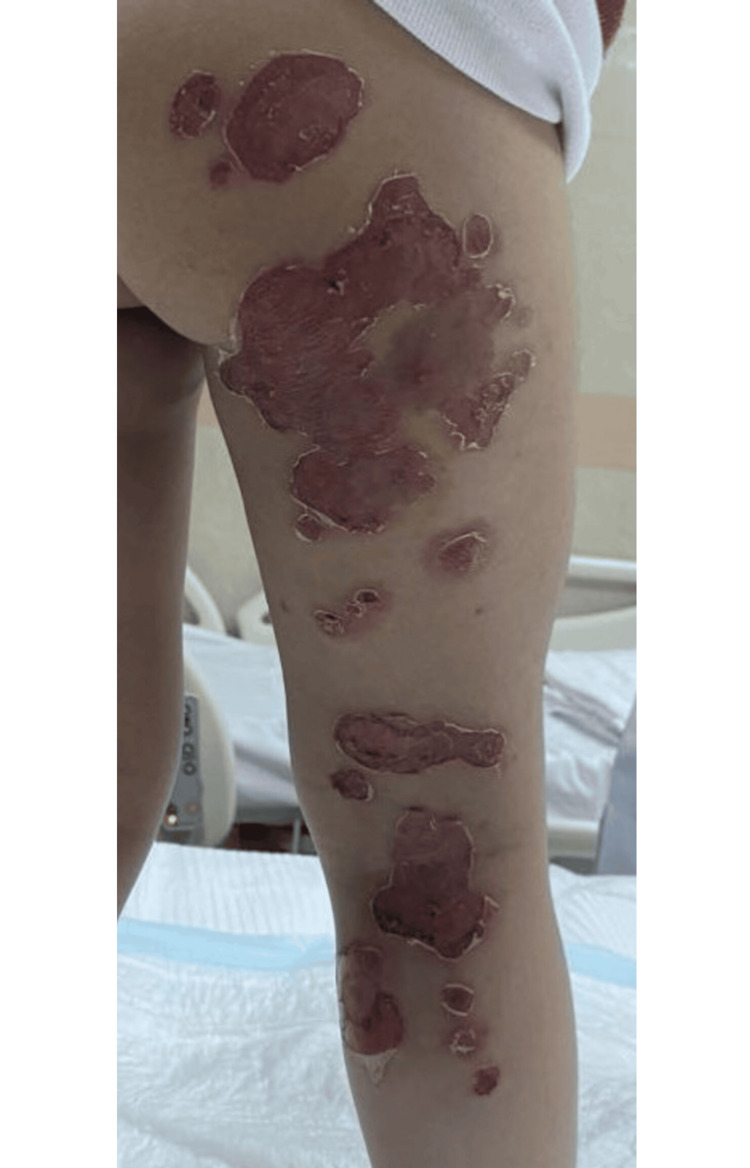
Lesions on Admission Flaccid bullae and superficial erosions involving the right buttock and posterior thigh at presentation. Multiple ruptured bullae with well-demarcated erosions and peripheral collarettes of scale are visible, consistent with bullous impetigo. The lesions show areas of serous crusting and partial desiccation without surrounding cellulitic erythema.

A previously healthy two-year-old girl presented to the pediatric emergency department with a two-week history of gradually progressive flaccid, transparent bullae and superficial erosions with surrounding honey-colored crusts, predominantly involving the right lower limb and buttock. The lesions were severely pruritic and painful, accompanied by scattered erythematous papules (Figure 1). There was no mucosal involvement, lymphadenopathy, ocular or genital lesions, angioedema, or features of anaphylaxis. A focused blistering-disorder assessment was performed, and the Nikolsky sign was negative, supporting a localized process rather than a systemic exfoliative disorder. She did not report fever, fatigue, arthralgia, headache, gastrointestinal symptoms, or recent viral illness. Further history revealed that both of her siblings had similar but milder perioral lesions.

One week after the lesion onset, she was evaluated at a primary care clinic and prescribed topical betamethasone, fusidic acid, and oral clarithromycin; however, the use of topical corticosteroids is generally not recommended in suspected impetigo, as they may mask infection progression or delay appropriate antimicrobial management. Despite full compliance, no clinical improvement was noted, prompting presentation to the emergency department. She was subsequently admitted for further evaluation and management.

Routine laboratory investigations-including complete blood count, erythrocyte sedimentation rate, C-reactive protein, renal function, and liver profile-were all within normal limits (Table 1). Methicillin-resistant *S. aureus* (MRSA) screening swabs from the groin, axilla, and nasal cavity, as well as a skin swab for culture and sensitivity, were negative (Table 2). Although cultures from intact bullae fluid are known to provide higher diagnostic yield in bullous impetigo, no intact bullae were available at the time of assessment, and thus, swab cultures were performed instead.

**Table 1:** Routine Laboratory Investigations on Admission Demonstrating Normal Inflammatory and Metabolic Parameters Complete blood count, erythrocyte sedimentation rate, C-reactive protein, renal function, and liver profile were all within normal limits at the time of presentation. WBC: white blood cell; RBC: red blood cell; ALT: alanine transaminase; AST: aspartate aminotransferase; CRP: C-reactive protein; ESR: erythrocyte sedimentation rate

Test	Value	Reference range (unit)
WBC	11	5-15 (10^9^/L)
RBC	4.8	4-5.2 (10^12^/L)
Hemoglobin	130	110-140 (g/L)
Platelet count	350	200-490 (10^9^/L)
Lymphocytes	7.7	6-9 (10^9^/L)
Creatinine	28	11-34 (µmol/L)
Sodium	138	136-144 (mmol/L)
Potassium	4.2	3.6-5.1 (mmol/L)
Total protein	68	57-80 (g/L)
Albumin	46	35-52 (g/L)
Total bilirubin	11.6	5-21 (µmol/L)
ALT	15	3-35 (U/L)
AST	18	3-35 (U/L)
CRP	6	0-8 (mg/L)
ESR	14	0-20 (mm/hr)

**Table 2:** Skin Swab Culture Results Obtained on Admission Skin swab culture from the affected area showed no growth of pathogenic organisms. This result was interpreted in the context of prior antimicrobial exposure, as the patient had already completed several days of topical and systemic antibiotics, which may have reduced culture yield.

Microbiology	
Specimen	Skin swab culture
Culture yields	No growth after 5 days of incubation

**Figure 2:**
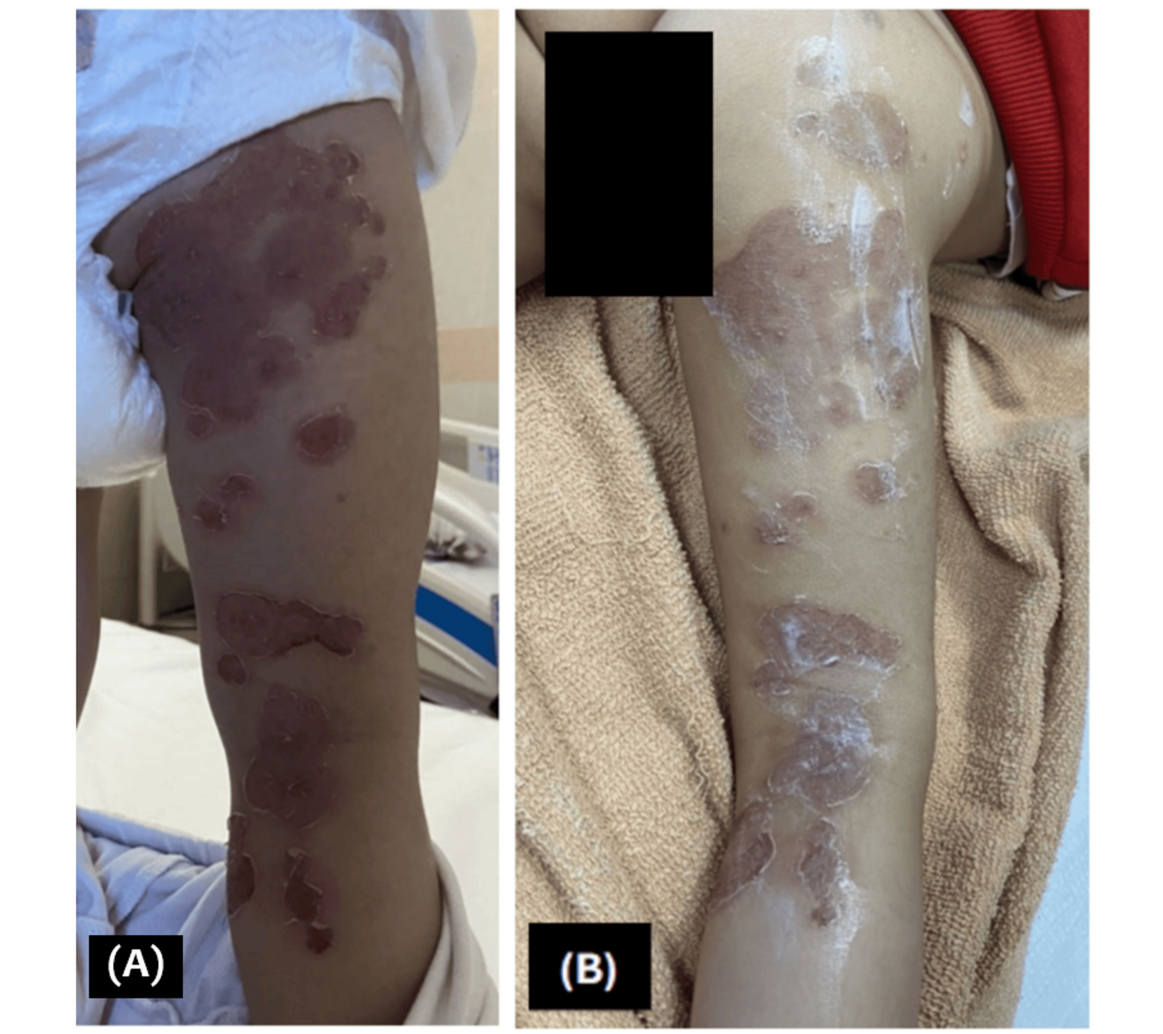
Progressive Improvement of Bullous Impetigo Lesions Following Initiation of Systemic and Topical Therapy (A) Day 2 post-treatment showing decreased crusting, reduced erythema, and early re-epithelialization of previously eroded plaques. (B) Day 3 post-treatment demonstrating continued healing with contraction of erosions and fading erythema.

On admission, empiric therapy with intravenous cefazolin (250 mg per dose) was initiated, along with topical fusidic acid and emollients. Dermatology evaluation supported the clinical diagnosis of bullous impetigo, and the same treatment regimen was continued. Over the following days, the patient demonstrated progressive improvement, with a reduction in pruritus and crusting (Figure 2). After three days of inpatient therapy, she was discharged on oral cephalexin (250 mg) for one week in addition to continued topical fusidic acid and emollients.

## Discussion

Impetigo is the most common superficial bacterial infection in children, particularly those between two and five years of age. It is a highly contagious infection of the superficial epidermis and remains a significant cause of morbidity in pediatric populations worldwide [1].

Two clinical subtypes are recognized: non-bullous and bullous impetigo. Non-bullous impetigo accounts for approximately 70% of cases and is caused by *S. aureus*, *S. pyogenes*, or both. Bullous impetigo represents the remaining cases and is almost exclusively attributed to exfoliative toxin-producing *S. aureus* [2].

Bullous impetigo is characterized by small vesicles that enlarge into flaccid, superficial bullae measuring up to 2 cm in diameter. The bulla contents are initially clear but may become turbid or purulent over time. Because of their fragile nature, the bullae rupture easily, leaving behind shallow erosions bordered by a collarette of scale and a thin, brown crust. Lesions commonly arise on previously normal skin, with a predilection for the trunk, extremities, and moist intertriginous areas such as the axillae, neck folds, and diaper region. Systemic symptoms are uncommon in bullous impetigo, occurring in fewer than 10%-15% of cases, and most patients appear well. However, pruritus, localized discomfort, and regional lymphadenopathy may occur. Mild leukocytosis can also be present, although surrounding erythema is typically minimal or absent [3].

Primary impetigo results from direct bacterial invasion of intact, healthy skin, whereas secondary impetigo arises in areas of disrupted epidermal barrier such as abrasions, insect bites, dermatitis, or pre-existing dermatoses. Non-bullous impetigo may be caused by *S. aureus*, *S. pyogenes*, or both. In contrast, bullous impetigo is exclusively associated with exfoliative toxin-producing strains of methicillin-sensitive *S. aureus* (MSSA) and MRSA. These strains secrete exfoliative toxins A, B, and D, which specifically target desmoglein-1, a cadherin-type desmosomal protein responsible for keratinocyte adhesion in the upper epidermis. Exfoliative toxin A is the predominant toxin identified in pediatric bullous impetigo. Proteolytic cleavage of desmoglein-1 induces loss of cohesion within the granular layer, resulting in superficial intraepidermal blister formation characteristic of bullous impetigo. The localized nature of toxin activity distinguishes bullous impetigo from staphylococcal scalded skin syndrome (SSSS), where systemic dissemination of toxins leads to widespread epidermal cleavage [3].

Several risk factors predispose children to impetigo, including warm and humid climates, poor hygiene, close physical contact, overcrowded living conditions, and lower socioeconomic status. Skin barrier-compromising conditions such as atopic dermatitis, scabies, and insect bites also increase susceptibility. Children aged two to five years are particularly vulnerable due to immature immunity and high rates of close contact exposures. Attendance at daycare centers or nurseries is also a recognized risk factor due to frequent interpersonal contact and shared environments [4].

The diagnosis of bullous impetigo is primarily clinical, based on the presence of flaccid bullae, superficial erosions, and characteristic collarettes of scale. In most cases, history and examination are sufficient. Gram stain and bacterial culture of bulla fluid, pus, or exudate may aid in differentiating *S. aureus* from *S. pyogenes* and are recommended when MRSA is suspected or when treatment failure occurs. Skin biopsy is generally unnecessary but may be considered when the presentation is atypical, when expected improvement does not occur despite appropriate antimicrobial therapy, or when the morphology raises concern for immunobullous diseases, viral infections, or other blistering conditions requiring histopathologic confirmation. Histopathologic examination of bullous impetigo typically reveals a subcorneal or granular layer intraepidermal blister. The blister cavity contains neutrophils, fibrin, and occasional acantholytic keratinocytes. Additional features may include spongiosis, papillary dermal edema, and a superficial perivascular inflammatory infiltrate composed of lymphocytes and neutrophils. These findings help distinguish bullous impetigo from immunobullous disorders and other blistering conditions. The differential diagnosis of bullous impetigo includes both infectious and non-infectious entities. Conditions that may mimic bullous impetigo include allergic contact dermatitis, burns, and early bullous pemphigoid. Dermatitis herpetiformis and erythema multiforme may also present with vesiculobullous lesions but typically demonstrate distinct distribution patterns and associated symptoms. In young children, early varicella lesions or inflamed molluscum contagiosum papules may occasionally resemble the initial vesiculobullous changes of impetigo and should be considered. For non-bullous presentations, the differential diagnosis includes contact dermatitis, scabies, dermatophytosis (tinea corporis or capitis), herpes simplex virus infection, and eczema herpeticum. Careful assessment of lesion morphology, distribution, and associated features is essential for accurate distinction among these entities [3].

Although impetigo is a self-limited infection that typically resolves within two weeks without scarring, treatment is recommended to reduce transmission, hasten clinical improvement, and prevent complications. Gentle cleansing of affected areas and removal of crusts with warm water or antiseptic washes, such as chlorhexidine, are commonly advised supportive measures.

Topical antibiotics are considered first-line therapy for localized disease. Agents such as mupirocin, retapamulin, or fusidic acid demonstrate excellent efficacy against *S. aureus* and *S. pyogenes*. Topical therapy is generally preferred for patients with a limited number of lesions, particularly in non-bullous presentations [5].

Systemic antibiotics are indicated for patients with extensive lesions, bullous disease, multiple affected body sites, or associated systemic symptoms, or when topical therapy is impractical. Oral cephalexin and dicloxacillin are recommended first-line options due to their reliable activity against MSSA and streptococcal species. A treatment duration of seven days is commonly sufficient. Macrolide antibiotics, such as erythromycin or clarithromycin, may be used in cases of penicillin allergy, although rising resistance limits their utility in some regions.

When MRSA is suspected or confirmed, alternative agents such as clindamycin, doxycycline (in children ≥8 years), or trimethoprim-sulfamethoxazole should be considered, guided by local resistance patterns. Culture and sensitivity testing is useful for optimizing therapy in refractory cases or areas with high MRSA prevalence [6].

Impetigo is generally a self-limited condition with an excellent prognosis, and most children recover without scarring. However, complications may occur, particularly in untreated, widespread, or high-risk cases. Post-infectious sequelae are uncommon but clinically significant. Non-bullous impetigo caused by *S. pyogenes* can lead to post-streptococcal glomerulonephritis (PSGN), which typically presents one to three weeks after infection. PSGN may manifest with hematuria, edema, and hypertension, although many cases remain mild or subclinical. Importantly, antibiotic treatment does not reduce the risk of PSGN. Local complications include cellulitis, lymphangitis, and regional lymphadenitis. More invasive infections such as osteomyelitis, septic arthritis, pneumonia, or bacteremia are rare but have been reported, particularly in young children or immunocompromised individuals [7].

Bullous impetigo, caused by exfoliative toxin-producing *S. aureus*, may progress to SSSS when the toxins disseminate systemically. SSSS is characterized by diffuse tender erythema, superficial blistering, and exfoliation resembling a scald injury, often accompanied by fever and irritability. This represents a dermatologic emergency requiring prompt systemic therapy. Overall, with timely treatment, the prognosis of impetigo is excellent, and serious complications remain uncommon [3].

## Conclusions

This case describes a two-year-old girl with bullous impetigo who presented with progressive flaccid bullae and erosions localized to the right lower limb. She responded well to intravenous cefazolin followed by oral therapy, with rapid improvement and complete resolution of lesions. The case highlights the value of recognizing the characteristic clinical features of bullous impetigo and considering it in the differential diagnosis of pediatric vesiculobullous eruptions. Early clinical diagnosis helped avoid unnecessary investigations-such as routine skin biopsies, extensive viral testing, or repeated bacterial cultures-which are commonly overused in similar vesiculobullous presentations, and guided effective management.
